# Genetic Divergence between *Camellia sinensis* and Its Wild Relatives Revealed via Genome-Wide SNPs from RAD Sequencing

**DOI:** 10.1371/journal.pone.0151424

**Published:** 2016-03-10

**Authors:** Hua Yang, Chao-Ling Wei, Hong-Wei Liu, Jun-Lan Wu, Zheng-Guo Li, Liang Zhang, Jian-Bo Jian, Ye-Yun Li, Yu-Ling Tai, Jing Zhang, Zheng-Zhu Zhang, Chang-Jun Jiang, Tao Xia, Xiao-Chun Wan

**Affiliations:** 1 State Key Laboratory of Tea Plant Biology and Utilization, Anhui Agricultural University, Hefei, 230036, China; 2 Department of Applied Chemistry, School of Science, Anhui Agricultural University, Hefei, 230036, China; 3 School of Information & Computer, Anhui Agricultural University, Hefei, 230036, China; 4 BGI-Shenzhen, Shenzhen, 518083, China; National Institute of Plant Genome Research, INDIA

## Abstract

Tea is one of the most popular beverages across the world and is made exclusively from cultivars of *Camellia sinensis*. Many wild relatives of the genus *Camellia* that are closely related to *C*. *sinensis* are native to Southwest China. In this study, we first identified the distinct genetic divergence between *C*. *sinensis* and its wild relatives and provided a glimpse into the artificial selection of tea plants at a genome-wide level by analyzing 15,444 genomic SNPs that were identified from 18 cultivated and wild tea accessions using a high-throughput genome-wide restriction site-associated DNA sequencing (RAD-Seq) approach. Six distinct clusters were detected by phylogeny inferrence and principal component and genetic structural analyses, and these clusters corresponded to six *Camellia* species/varieties. Genetic divergence apparently indicated that *C*. *taliensis* var. *bangwei* is a semi-wild or transient landrace occupying a phylogenetic position between those wild and cultivated tea plants. Cultivated accessions exhibited greater heterozygosity than wild accessions, with the exception of *C*. *taliensis* var. *bangwei*. Thirteen genes with non-synonymous SNPs exhibited strong selective signals that were suggestive of putative artificial selective footprints for tea plants during domestication. The genome-wide SNPs provide a fundamental data resource for assessing genetic relationships, characterizing complex traits, comparing heterozygosity and analyzing putatitve artificial selection in tea plants.

## Introduction

Tea is one of the most popular non-alcoholic beverages and is consumed by more than one third of the world’s population due to its stimulant effects, attractive aroma, refreshing taste and health benefits [1]. The ancestors of the cultivated tea plants are native to Southwest China, and cultivated varieties are now grown in the majority of tropical and subtropical regions of the world. In these locations, tea is an economically important crop [2–5]. By far, the most commercially important variety of this evergreen woody crop is *Camellia sinensis* (L.) O. Kuntze, which belongs to the section *Thea* of the genus *Camellia* in the family *Theaceae*. *C*. *sinensis* includes two main varieties, i.e., *C*. *sinensis* var. *sinensis* and *C*. *sinensis* var. *assamica*.

Systematic studies of wild tea germplasm resources were initiated in 1980s and have identified numerous wild tea species that are native to the Yunnan province in Southwest China. The majority of wild tea plants are close relatives of *C*. *sinensis*, such as *C*. *tachangensis*, *C*. *taliensis* and *C*. *crassicolumna* and *C*. *gymnogyna* etc., all of which belong to section *Thea* [3, 5–6]. Although wild and cultivated varieties are monoecious, insectpollinated and self-incompatible species, according to Zhang [3] and Ming [5], diverse morphophysiological traits, such as the number of locules per ovary, the sizes of sepals and petals, the characters of leaves and pedicels etc., exist between wild and cultivated varieties. Especially, *C*. *tachangensis*, *C*. *taliensis* and *C*. *crassicolumna* have the features of the 5-locule ovaries, large sepals and petals, whearas *C*. *sinensis* has the features of 3-locule ovaries, small sepals and petals. The accession of *C*. *taliensis* var. *bangwei*, which was identified to be the only known semi-wild tea plant worldwide until now because it exhibited characteristics of both cultivated and wild tea plants based on evidence from previous morphological trait and karyotype analysis [7]. Diverse types of foliar sclereids were also detected in *C*. *sinensis* and its wild relatives in section *Thea* [8]. Although *C*. *sinensis* is currently the only mass-cultivated and commercially viable species, the use of other wild relatives as beverages is being explored [9]. Most importantly, wild tea plants are reservoirs of genetic diversity that provide materials for molecular genetic studies and breeding programs that aim to engineer variants with improved yield, disease resistance and tolerance to different environmental conditions [10].

*C*. *sinensis* and its wild relatives in the section *Thea* possess large genomes of 2.2–4.0 Gb [11–12] that exhibit high heterozygosity due to genetic barriers such as self-incompatibility and the depression of inbreeding. Genomic information is currently limited, which hinders molecular genetic studies; however, a few molecular markers have been developed to study the genetic diversity of and relationships between tea cultivars and wild relatives using approaches such as amplified fragment length polymorphism (AFLP)[13–14], random amplified polymorphic DNA (RAPD) [13,15–16], simple sequence repeat (SSR) [17–19], inter-simple sequence repeat (ISSR) [20–21], internal transcribed spacer (ITS) [22] and chloroplast DNA loci [23–24] studies. However, these limited molecular markers cannot provide sufficient resolution for phylogenetic relationship inferences. With the advent of next-generation sequencing (NGS) technologies, two recent studies reported the chloroplast genomes and phylogenetic relationships of a number of *Camellia* species and varieties [25–26]. Because chloroplast genome data are limited in the capacity to resolve phylogenetic relationships in species undergoing rapid evolution [27–28], it is necessary to develop more genome data resources, including novel and high-throughput genomic markers, to facilitate genome-scale molecular genetics research in cultivated and wild teas.

As the most abundant type of sequence variations distributed within genomes, SNPs can be easily identified by sequence comparisons of both alleles of a diploid genome, expressed sequence tags (ESTs), and unigenes derived from transcriptome sequences [29–31]. Due to their low cost, high genotyping efficiency, genome-wide coverage and analytical simplicity [32], SNPs have rapidly become the preferred marker type for comparative genetic studies. In *C*. *sinensis*, totals of 818 and 1,786 EST-SNPs mined from ESTs and mRNA nucleotide sequences in GenBank, respectively, were used to analyze the genetic relationships between varieties [33–34]. Recently, the first reference genetic map of *C*. *sinensis* was constructed using 6,042 SNP markers from an F1 mapping population of tea cultivars through a specific-locus amplified fragment sequencing (SLAF-seq) approach [35]. In contrast, few genomic SNPs have been identified in the wild relatives of the genus *Camellia* and applied to the study of genetic diversity and the relationships between cultivated and wild teas.

The high-throughput NGS technologies have proven useful for the large-scale discovery of genome-wide SNPs in complex genomes [36]; these technologies include RAD-seq [37], complexity reduction of polymorphic sequences (CRoPS) [38], reduced representation libraries (RRLs) [39], genotyping by sequencing (GBS) [40], sequence-based genotyping (SBG) [41] and SLAF-seq [35], and have been widely used for genotyping and the development of genome-scale genetic markers. Common to all of these approaches is the initial usage of restriction enzymes and subsequent sequencing of a small section of the genome to reduce the complexity of the target DNA. RAD-Seq, which was developed to identify polymorphic variants in genomic regions adjacent to restriction enzyme digestion sites [37, 42], has proven to be particularly suitable for species that lack a published genome sequence [43–45] and has provided genome-scale SNP data that have successfully revealed information for phylogenetic inferences in *Pedicularis* [46], temperate bamboos [47] and Chinese bayberry [48], population genetics [49–50], species identification [51–52], species evolution [53] and phylogenomics [54–55]. Additionally, RAD-Seq can also be utilized for association mapping [56] and genetic mapping [42, 57].

In this study, we used RAD-Seq for rapid, cost-effective, high-throughput SNP discovery in 18 cultivated and wild tea accessions belonging to the section *Thea* of the genus *Camellia*. Using the identified genomic SNPs, we constructed the phylogenetic relationships among the different accessions on a genome-wide scale. Furthermore, genic SNPs related to functional genes and SNPs that have been under selective pressure during domestication were also discussed.

## Results and Discussion

### High-throughput RAD sequencing and *de novo* SNP discovery

A total of 18 tea accessions of *Camellia sinensis* and its wild relatives from the genus *Camellia* (Table 1) were used for the construction of RAD libraries and single-ended sequencing on Illumina Hiseq 2000 platform. After trimming the barcodes, quality filtering and cleaning of the raw reads, a total of 52.90 gigabase pairs (GB) of high-quality clean reads with a length of 41 nucleotides (nt) carrying 5 nt of the *EcoR*I recognition site and 36 nt of potentially variable sequence were generated (93.2% of the raw data, 1.71 GB to 4.23 GB for each accession, with an average of 2.94 GB per accession; Table 2 and S1 Table). All of the RAD data have been deposited in Short Read Archive (SRA) of GenBank under accession SRP030678. Using the Stacks pipeline [56], we initially obtained 18,290,143 candidates of the RAD tag loci from all of the accessions and 5,674,749 heterozygous loci identified by genotyping (an average of 315,264 for each accession; Table 2 and S1 Table). Comparisons of these RAD tag loci between all accessions ultimately revealed a total of 15,444 bi-allelic SNP loci shared by 14 or more accessions (Table 2, S2 and S3 Tables), with an average sequencing depth of approximetely 42-fold per nucleotide position, which corresponds to an average RAD genomic size of 0.56 megabase pairs (MB) (Table 2 and S3 Table). Of the 15,444 SNPs, 9,227 (59.7%) were observed to be transitions (C/T or G/A), and 6,217 (40.3%) were transversions (C/T, A/G, C/A, or T/G; S1 Fig), and the transition/transversion ratio (TI/TV) was 1.48, which is lower than the previously reported 2.0 for EST-SNPs in tea [33], and similar to those of grapes (1.46) [59] and potatoes (1.5) [60] and higher than that of soybeans (0.92) [61]. The frequency of C/T alleles was the highest (4,695, 30.4% of all alleles; S1 Fig), which agree with the observations in tea ESTs [33] and is similar to those of beans [62], maize [63] and *Citrus spp*. [64–65].

**Table 1:** The 18 tea accessions of *Camellia sinensis* and its wild relatives used in this study.

Code	Accession Name	Species/Varieties	Sample Type	Sampling Location
*Ctl-1*	Bada 1	*C*. *taliensis*	wild	Menghai country, Yunnan province
*Ctl-2*	Bada 4	*C*. *taliensis*	wild	Menghai country, Yunnan province
*Ctl-3*	Daxueshan	*C*. *taliensis*	wild	Shuangjiang country, Yunnan province
*Ccc-1*	Daweishan 1	*C*. *crassicolumna*	wild	Tai Wai Mountain National Nature Reserve, Pingbian country, Yunnan province
*Ccc-2*	Daweishan 2	*C*. *crassicolumna*	wild	Tai Wai Mountain National Nature Reserve, Pingbian country, Yunnan province
*Ccc-3*	Daweishan 4	*C*. *crassicolumna*	wild	Tai Wai Mountain National Nature Reserve, Pingbian country, Yunnan province
*Ccc-4*	Daweishan 5	*C*. *crassicolumna*	wild	Tai Wai Mountain National Nature Reserve, Pingbian country, Yunnan province
*Ctg*	Fuyuan	*C*. *tachangensis*	wild	Fuyuan country, Yunnan province
*Ctb*	Bangwei	*C*. *taliensis* var. *bangwei*	semi-wild	Shuangjiang country, Yunnan province
*Csa-1*	Nanruoshan 1	*C*. *sinensis* var. *assamica*	cultivated	Nanruo Moutain, Menghai country, Yunnan province
*Csa-2*	Nanruoshan 2	*C*. *sinensis* var. *assamica*	cultivated	Nanruo Moutain, Menghai country, Yunnan province
*Csa-3*	Yunkang 10	*C*. *sinensis* var. *assamica*	cultivated	Tea Research Institute of Yunnan Academy of Agricultural Science
*Css-1*	Shuchazao	*C*. *sinensis* var. *sinensis*	cultivated	Agricultural plantations of Anhui Agricultural University
*Css-2*	Longjing 43	*C*. *sinensis* var. *sinensis*	cultivated	Agricultural plantations of Anhui Agricultural University
*Css-3*	Anhui 1	*C*. *sinensis* var. *sinensis*	cultivated	Tea Research Institute of Anhui Academy of Agricultural Science
*Css-4*	Tieguanyin	*C*. *sinensis* var. *sinensis*	cultivated	Tea Research Institute of Fujian Academy of Agricultural Science
*Css-5*	Fudingdabai	*C*. *sinensis* var. *sinensis*	cultivated	Tea Research Institute of Yunnan Academy of Agricultural Science
*Css-6*	F1individual from “Yunkang 10 × Fudingdabai”	*C*. *sinensis* var. *sinensis*	cultivated	Tea Research Institute of Yunnan Academy of Agricultural Science

**Table 2:** Summary of the RAD sequencing and *de novo* SNP discovery in the 18 tea accessions.

Category	Total counts	Mean counts	Total data size (MB)	Mean data size (MB)	Average depth (X)
Raw reads	1,305,108,148	72,506,008	56,775.6	3,154.2	-
Clean reads	1,290,292,866	71,682,937	52,902.0	2,939.0	-
RAD tag loci	18,290,143	1,016,119	749.9	41.7	70.4
Heterozygous RAD tag loci	5,674,749	315,264	232.7	12.9	-
Bi-allelic SNPs identified from the 18 tea accessions	15,444	13,669	0.63	0.56	41.5

### Genetic relationship between cultivated and wild accessions

**Fig 1:**
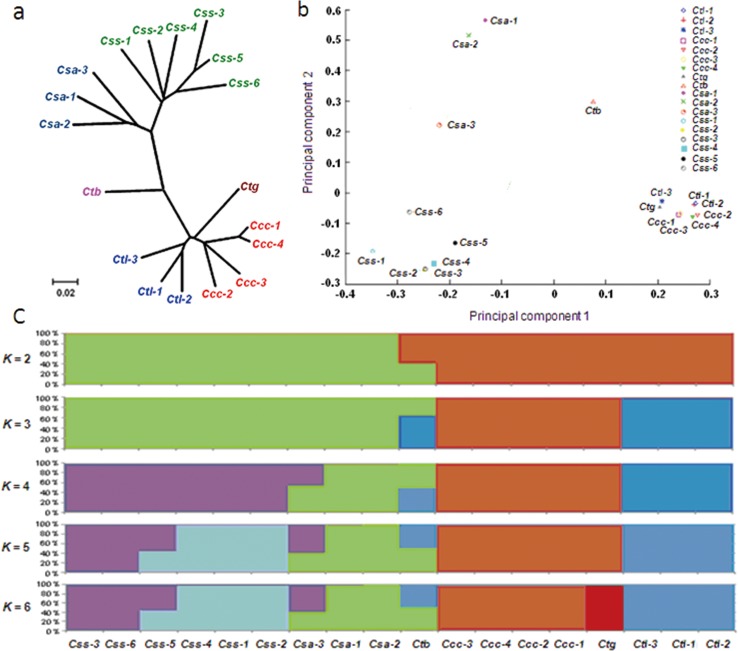
Neighbor-joining phylogenetic tree, plot of the principle component analysis (PCA) and genetic structures for the 18 tea accessions. (a) Neighbor-joining phylogenetic tree based on 15,444 identified SNPs with bootstrap values calculated from 1,000 trees. (b) Principal component analysis of the 18 tea accessions. (C) Genetic structure of the 18 tea accessions. Different inferred populations are distinguished by different colors. Each accession is indicated by a vertical bar, and the length of each colored section in each vertical bar represents the proportion from ancestral populations.

To examine the genetic relationships between cultivated and wild accessions, a neighbor-joining phylogenetic analysis [66–67] and principle component analysis (PCA) [68] were conducted using the 15,444 genomic SNPs. Based on the genetic distances of the genotyped SNPs, the 18 accessions were clustered into six clades. The *Css* and *Csa* clades contained six cultivars of *C*. *sinensis* var. *sinensis* (*Css-1*, *Css-2*, *Css-3*, *Css-4 Css-5*, and *Css-6*) and three cultivars of *C*. *sinensis* var. *assamica* (*Csa-1*, *Csa-2* and *Csa-3*). Another four clades (*Ccc*, *Ctl*, *Ctb* and *Ctg*) were composed of wild accessions. The *Ctb* accession from *C*. *taliensis* var. *bangwei* formed a cluster that was distinct from the other *C*. *taliensis* accessions, and the *Ctg* branch contained the sole *Ctg* accession from *C*. *tachangensis* (Fig 1A). PCA using the first and second eigenvectors identified six clusters, i.e., *Css*, *Csa*, *Ccc*, *Ctl*, *Ctb* and *Ctg* groups, which were consistent with the phylogenetic clades. The PCA plot illuminated that the *Css*, *Csa* and *Ctb* clusters were more disperse than the *Ccc*, *Ctl* and *Ctg* clusters (Fig 1B).

The estimation of the individual ancestries was performed based on maximum likelihood using the admixture proportions (*K* represents the number of inferred populations) from 2 to 6 provided by the FRAPPE program [69] (Fig 1C). For *K* = 2, a division was identified between the tested cultivated and wild accessions. Specifically, the *Ctb* accession displayed an admixture of cultivated and wild accessions. When *K* = 3, the *Ctl* group was distinguished from any other wild accession, and the *Ctb* accession appeared to share an ancestry with *Ctl*. At *K* = 4, the cultivated accessions were clearly divided into the *Csa* and *Css* groups (Fig 1C). The *Ctb* accession exhibited an admixture of *Ctl* and *Csa*. For *K* = 6, the *Ctg* accession was separated from the *Ccc* group within the wild accessions in contrast to the observations at *K* = 3. The three parallel analyses (phylogenetic, principle component and genetic structure analyses) provided comprehensive molecular evidence regarding the species boundaries between C. *sinensis* var. *sinensis*, *C*. *sinensis* var. *assamica*, *C*. *crassicolumna*, *C*. *taliensis*, *C*. *taliensis* var. *bangwei* and *C*. *tachangensis* in the section *Thea* of the genus *Camellia*.

Tea accessions belonging to *C*. *sinensis* var. *sinensis* and *C*. *sinensis* var. *assamica* were genetically distinct from the other four wild relatives/varieties in accordance with the chloroplast genomic data [26]. Although clearly divergent from the other accessions, the genetic relationship between *C*. *sinensis* var. *sinensis* and *C*. *sinensis* var. *assamica* was the closest. These accessions may have independently evolved from a common *C*. *sinensis* ancestor. Similarly, the three wild relatives, *C*. *taliensis*, *C*. *crassicolumna* and *C*. *tachangensis*, were found to be divergent but clustered tightly together. In addition, using HPLC analysis, we have detected the contents of catechins (flavan-3-ols), one kind of characteristic secondary metabolites contributing to tea quality [70], in the same wild and cultivated tea accessions as mentioned above. Quantitative analysis of the average contents of total catechins (non-galloylated catechins and their gallate esters) exhibited that those in cultivated tea varieties (averagely 170.95 mg·g^-1^ in *C*. *sinensis* var. *sinensis* and 277.38 mg·g^-1^ in *C*. *sinensis* var. *assamica*) were rather higher than those in wild varieties (averagely 28.87 mg·g^-1^ in *C*. *taliensis*, 16.14 mg·g^-1^ in *C*. *crassicolumna* and 44.25 mg·g^-1^ in *C*. *tachangensis*). Metabolomic analysis also identified eight compounds related to non-galloylated catechins and their gallate esters that were considered to be the candidate biomarkers contributing to the significant differences in the characteristics between cultivated and wild tea accessions (unpublished data). The phytochemical differentiation of cultivated and wild tea plants independently supported the genetic divergence of them inferred from RAD-Seq data. Interestingly, *Ctb* is the only known semi-wild or transient landrace that shared the characteristics of both the cultivated and wild varieties [7]. The average content of total catechins of *C*. *taliensis* var. *bangwei* was 114.98 mg·g^-1^, representing a median level between the wild and cultivated varieties. Consistently, our phylogenetic tree revealed that the landrace occupied a phylogenetic position between the wild and cultivated varieties, exhibiting closest relationship between *C*. *taliensis* and *C*. *sinensis* var. *assamica* (Fig 1A). As a potential admixture of *C*. *taliensis* and *C*. *sinensis* var. *assamica* (Fig 1C), we predicted that *Ctb* might be an interspecific hybrid of the two species.

## Heterozygosity

**Fig 2:**
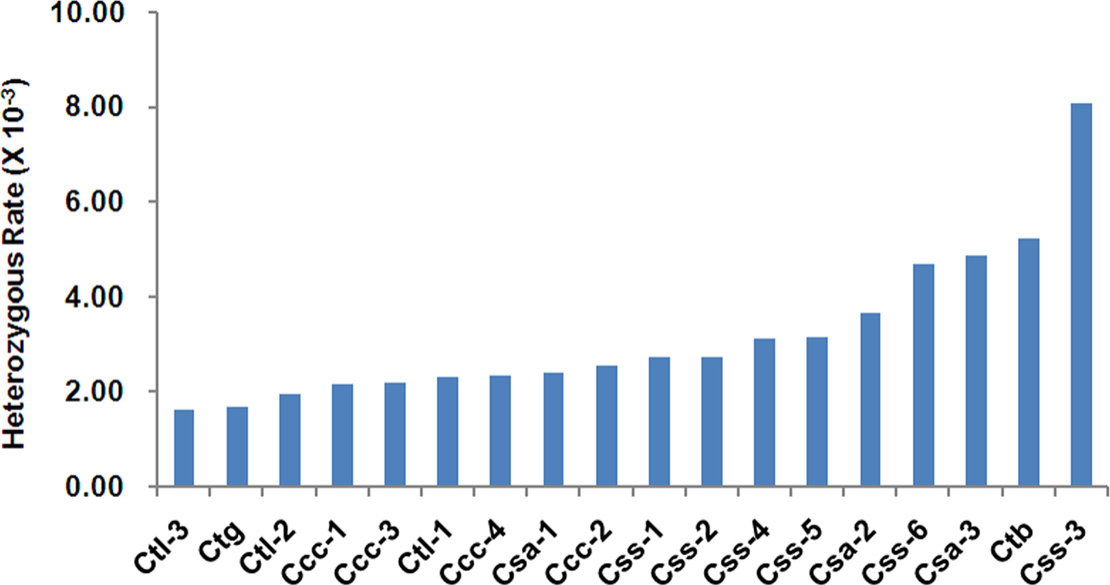
Heterozygosity levels of the 18 tea accessions. The heterozygous rates of 18 tested tea accessions were evaluated by calculating the ratio of the number of heterozygous SNPs to the length of the shared SNP-associated genome fragments from the RAD sequencing in each accession.

To investigate the heterozygous rates of the cultivated and wild tea accessions, we identified an average of 1,836 heterozygous SNPs per accession using the genotyping data of 15,444 bi-allelic SNPs, which reflected total average heterozygous rate of 3.2 per Kb across all of the 18 accessions (Fig 2 and S3 Table). Accession *Ctl-3* exhibited the lowest heterozygosity at 1.6 per Kb, and *Css-3* exhibited the highest at 8.1 per Kb. The heterozygous rates of *C*. *tachangensis*, *C*. *taliensis*, *C*. *crassicolumna*, *C*. *sinensis* var. *assamica*, *C*. *sinensis* var. *sinensis* and *C*. *taliensis* var. *bangwei* were1.7, 2.0, 2.4, 3.7, 4.1 and 5.2 per Kb, respectively (S2 Fig), suggesting that the cultivated accessions possessed greater heterozygosity than most of the tested wild accessions with the exception of *C*. *taliensis* var. *bangwei*.

The comparatively lower nucleotide variation within the wild accessions might be associated with lower rates of natural hybridization and introgression. As far as their distribution areas were concerned, most of the wild tea accessions are distributed within a narrow geographic environment (mainly in the Yunnan province) in areas with relatively small populations. Because the cultivars are planted northwards from their center of origin across vast geographical areas, self-incompatibility and long-term allogamy, domestication via hybridization, and climatic selection might have resulted in cultivars with broader genetic variation. The high heterozygosity in *C*. *taliensis* var. *bangwei* may be due to interspecific hybridization between the highly differentiated *C*. *taliensis* and *C*. *sinensis* var. *assamica* species. The introgression of wild relatives in tea breeding programs might help to maintain genetic variability in tea cultivars.

## Identification, functional analysis and validation of genic SNPs

**Fig 3:**
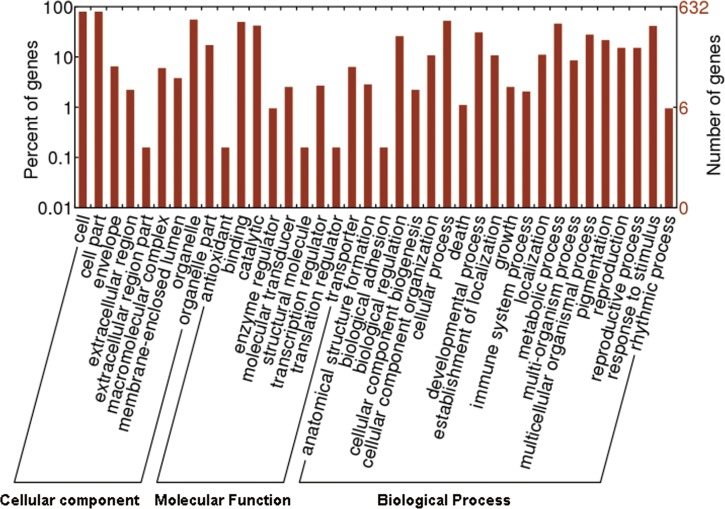
Gene Ontology classifications of the identified genic SNP-associated tea unigenes. GO terms were assigned to *C*. *sinensis* unigenes based on the top BLASTX hits against the NCBI *Arabidopsis* protein database. The GO terms were classified into three main GO categories (i.e., biological process, cellular component, molecular function) that included 38 sub-categories. The left y-axis indicates the proportion of genes in the main category, and the right y-axis indicates the number of genes in the same category.

The resultant 15,444 bi-allelic SNPs comprised gene-derived (genic) SNPs and non-genic SNPs. Genic SNPs, representing potential function-related single nucleotide variants, are helpful in understanding genetic drift, mutations and migrations in natural and cultivated tea populations, and are particularly valuable for characterizing genes associated with complex traits [71–72]. Genic SNPs were identified via comparisons with the tea transcriptome dataset (127,094 unigenes) of *C*. *sinensis* cv. *Longjing43* [73] using BLASTN with an E-value cut-off of 1e-5 and an allowed maximum mismatch of one. The alignments revealed 1,521 SNP-associated unigenes (S4 Table) in tested the tea accessions. Of these, a total of 1,058 tea unigenes (69.5% of 1,521) were annotated by alignments against the NCBI *Arabidopsis* protein dataset using BLASTX with an E-value threshold of 1e-5 (S5 Table). Functional analysis identified 632 tea genes (41.6% of 1,521) that were assigned to 3,230 Gene Ontology (GO) terms (Fig 3 and S6 Table) [74] using BLAST2GO [75], which were summarized into three main GO categories of “biological process” (2,095, 64.9%), “cellular component” (1,309, 49.1%), and “molecular function” (662, 20.4%; Fig 3 and S7 Table). The six major sub-categories of the biological process cluster were “cellular process” (GO: 0009987), “metabolic process” (GO: 0008152), “response to stimulus” (GO: 0050896), “developmental process” (GO: 0032502), “multicellular organismal process” (GO: 0032501) and “biological regulation” (GO: 0065007; Fig 3 and S7 Table). Three sub-categories of “cell” (GO: 0005623), “cell part” (GO: 0044464) and “organelle” (GO: 0043226) dominated the cellular component cluster, and the top two sub-categories in the molecular function cluster were “binding functions” (GO: 0005488) and “catalytic functions” (GO: 0003824; Fig 3 and S7 Table). A total of 24 unigenes were identified in secondary metabolic processes, including the sub-clusters of “phenylpropanoid metabolic process” (GO:0009698; including 12 unigenes invloved in phenylpropanoids and flavonoids metabolism; Table 3) and “terpenoid metabolic process” (GO:0006721; 7 unigenes; Table 3), which are important for detrmining tea quality [73]. Especially, the SNPs involved in phenylpropanoids and flavonoids metabolism may contribute to the variations of total catechins contents between wild and cultivated tea vareities.

**Table 3:** Genic SNP-associated tea unigenes involved in secondary metabolic processes.

**Tag ID**	**SNP**	Unigene ID	GO Category	GO Sub-category
Tea_307897	G/T	Singletons19599	phenylpropanoid metabolic process (GO:0009698)	phenylpropanoid metabolic process (GO:0009698)
Tea_301133	C/T	Singletons22060	phenylpropanoid metabolic process (GO:0009698)	phenylpropanoid metabolic process (GO:0009698)
Tea_300576	G/T	Singletons22067	phenylpropanoid metabolic process (GO:0009698)	phenylpropanoid metabolic process (GO:0009698)
Tea_303052	C/G	Singletons22068	phenylpropanoid metabolic process (GO:0009698)	phenylpropanoid metabolic process (GO:0009698)
Tea_304463	A/C	Singletons122210	phenylpropanoid metabolic process (GO:0009698)	phenylpropanoid biosynthetic process (GO:0009699)
Tea_303755	A/T	Singletons2015	phenylpropanoid metabolic process (GO:0009698)	phenylpropanoid biosynthetic process (GO:0009699)
Tea_307337	A/G	Singletons54227	phenylpropanoid metabolic process (GO:0009698)	phenylpropanoid biosynthetic process (GO:0009699)
Tea_296329	A/C	Singletons47964	phenylpropanoid metabolic process (GO:0009698)	flavonoid biosynthetic process (GO:0009813)
Tea_299422	C/T	Singletons49039	phenylpropanoid metabolic process (GO:0009698)	flavone biosynthetic process (GO:0051553)
Tea_300330	C/T	Singletons78302	phenylpropanoid metabolic process (GO:0009698)	flavone biosynthetic process (GO:0051553)
Tea_293997	A/G	Singletons51245	phenylpropanoid metabolic process (GO:0009698)	anthocyanin biosynthetic process (GO:0009718)
Tea_287303	C/G	Singletons16234	phenylpropanoid metabolic process (GO:0009698)	ignin metabolic process (GO:0009808)
Tea_301914	A/G	Singletons44363	terpenoid metabolic process (GO:0006721)	terpenoid metabolic process (GO:0006721)
Tea_298657	A/G	Singletons45405	terpenoid metabolic process (GO:0006721)	diterpenoid metabolic process (GO:0016101)
Tea_307068	A/T	Singletons26950	terpenoid metabolic process (GO:0006721)	sesquiterpenoid metabolic process (GO:0006714)
Tea_300741	A/C	Singletons33217	terpenoid metabolic process (GO:0006721)	sesquiterpenoid metabolic process (GO:0006714)
Tea_296981	G/T	Singletons50061	terpenoid metabolic process (GO:0006721)	tetraterpenoid metabolic process (GO:0016108)
Tea_304614	C/T	Singletons7787	terpenoid metabolic process (GO:0006721)	tetraterpenoid metabolic process (GO:0016108)
Tea_300741	A/C	Singletons33217	terpenoid metabolic process (GO:0006721)	tetraterpenoid metabolic process (GO:0016108)
Tea_301014	A/C	Singletons25297	phytochelatin metabolic process (GO:0046937)	regulation of flavonoid biosynthetic process (GO:0009962)
Tea_288785	A/G	Singletons30505	phytochelatin metabolic process (GO:0046937)	regulation of flavonoid biosynthetic process (GO:0009962)
Tea_301670	A/G	Singletons114182	glycosinolate metabolic process (GO:0019757)	glucosinolate catabolic process (GO:0019762)
Tea_307337	A/G	Singletons54227	glycosinolate metabolic process (GO:0019757)	glucosinolate catabolic process (GO:0019762)
Tea_306731	A/T	Singletons37370	glycosinolate metabolic process (GO:0019757)	glycosinolate biosynthetic process (GO:0019758)
Tea_304888	A/G	Singletons124304	alkaloid metabolic process (GO:0009820)	nicotinamide metabolic process (GO:0006769)
Tea_308736	C/T	Singletons15417	indole phytoalexin metabolic process (GO:0046217)	indole phytoalexin biosynthetic process (GO:0009700)

Additionally, we identified 453 genic SNPs that were located in the coding sequences of unigenes. Of these genic variations, 238 were non-synonymous substitutions, and 215 were synonymous (S8 Table). The ratio of non-synonymous to synonymous substitutions (dN/dS) was 1.1, which is similar to that of the rice genome (dN/dS = 1.2) [76], but higher than that of *Arabidopsis* (dN/dS = 0.8) [77]. The non-synonymous SNP-associated unigenes were grouped into 31 GO clusters, including 7 sub-clusters in the cell component cluster, 7 sub-clusters in the molecular function cluster and 17 sub-clusters in the biological process cluster (S3 Fig), which was indicative of invlovements in growth, development, regulation and stress resistance in tea.

**Fig 4:**
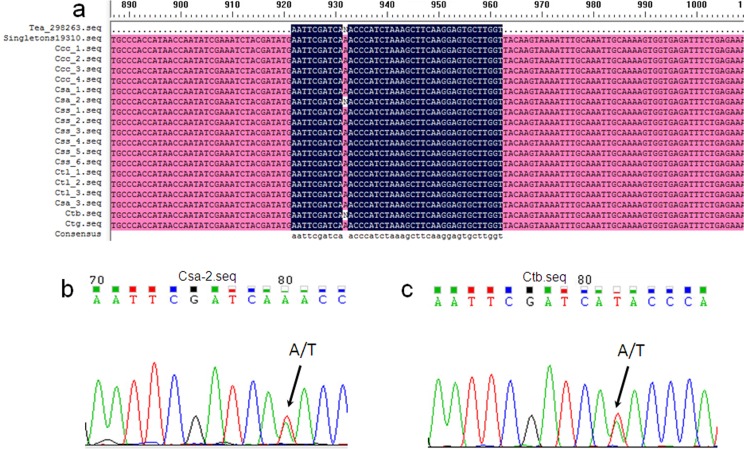
Validation of SNP identification and genotyping of the Tea_298263 SNP locus in the 18 tea accessions by PCR-based sequencing. (a) Flanking sequences adjacent to SNP loci obtained from Sanger sequencing were aligned against tag sequences containing SNP loci from RAD-Seq data and unigene Singletons19310 based on the top BLAST hits of the consensus tag sequences from *C*. *sinensis* var. *Longjing43* transcriptome [73] using DNAMAN software. N in the RAD tag sequence represents the SNP locus, which indicates the heterozygous genotypes in the SNP loci of the accessions *Csa-2* and *Ctb*. (b) Confirmation of the heterozygous genotypes (A/T) of the Tea_298263 SNP locus in accession *Csa-2* by Sanger sequencing. (C) Confirmation of the heterozygous genotype (A/T) of the Tea_298263 SNP locus in accession *Ctb* by Sanger sequencing.

To assess the accuracy of genic SNP identification and RAD-Seq-based genotyping analysis, we randomly selected 50 genic SNP loci from 900 genotypes across all of the 18 tested accessions to conduct PCR-based sequencing using SNP loci-specific primers (S9 Table). We found that these 50 SNP loci comprised 805 genotypes and 95 cases of missing data. A total of 767 PCR products corresponding to the 805 genotypes were successfully sequenced. The alignments of the sequences of the PCR products to the RAD-Seq data revealed consistency in 732 of the 805 genotypes (90.9%) between the two methods (Fig 4 and S10 Table). Over 90% (47/50) of the SNP loci derived from the RAD-Seq approach were therefore confirmed by this sampling analysis. Specifically, of the 50 randomly selected SNP loci, 7 were associated with genes involved in secondary metabolism processes (S10 Table). Among the 126 genotypes of the 7 loci, 99 of the 117 genotypes (84.6%) were consistent with those from the RAD-Seq data. As mentioned above, significant differences in flavonoid content (especially catechins and their gallate esters and anthocyanins) were apparent between the cultivated and wild accessions from phytochemical analysis. The observed single nucleotide mutations in the structural and regulatory genes involved in phenylpropanoid, flavonoid and anthocyanin metabolic processes might contribute to these secondary metabolite differences.

## Putative Selective Footprints during Tea Domestication

To identify the putative selective footprints of tea domestication, we calculated the divergence statistic π and the loss of diversity (LOD) [78] between the wild and cultivated groups based on the 15,444 SNPs. Only RAD tags containing SNP loci with a maximum LOD of 1 were treated as putative indicators of artificial selection. A total of 644 SNPs in the corresponding RAD tags were identified as subject to strong artificial selection (Table 4 and S11 Table). These SNP loci exhibited genetic diversity within the wild accessions (π_wild_ = 0.13 to 0.57) but had a fixed genotype at each locus in the cultivated accessions (π_cultivar_ = 0). Transitions and transversions accounted for 60.1% and 39.9%, respectively. We suggested that the loss of heterozygosity in the 644 SNP loci was probably due to the selection pressures of tea domestication.

**Table 4:** Tea SNPs that were subjected to strong selective pressures during domestication.

SNP Type	Genotype of SNP locus in wild accessions	Genotype of SNP locus fixed in cultivars	Number of SNP loci fixed in cultivars	π_wild_	Average π_wild_	π_cultivar_	LOD	Pencentage (%)
**Transition**	R: (A/G)	A	101	0.13–0.57	0.29	0	1	15.7
	R: (A/G)	G	95	0.13–0.56	0.3	0	1	14.8
	Y: (C/T)	C	103	0.13–0.57	0.29	0	1	16.0
	Y: (C/T)	T	88	0.13–0.57	0.32	0	1	13.7
	Total	—	387	0.13–0.57	0.30	0	1	60.1
**Transversion**	W: (A/T)	A	42	0.13–0.57	0.30	0	1	6.5
	W: (A/T)	T	41	0.13–0.56	0.28	0	1	6.4
	M: (A/C)	C	40	0.13–0.56	0.25	0	1	6.2
	M: (A/C)	A	28	0.13–0.57	0.28	0	1	4.3
	K: (G/T)	G	31	0.13–0.56	0.33	0	1	4.8
	K: (G/T)	T	32	0.13–0.57	0.24	0	1	5.0
	S: (C/G)	C	23	0.13–0.58	0.34	0	1	3.6
	S: (C/G)	G	20	0.13–0.56	0.31	0	1	3.1
	Total	—	257	0.13–0.57	0.29	0	1	39.9
**Total SNPs**	—	—	644	0.13–0.57	0.29	0	1	100

Eighty-one of the 644 SNPs were located in genic regions. Correspondingly, the SNP-associated RAD tags exhibited the best alignments with *C*. *sinensis* cv. *Longjing43* unigenes [73]. We identified 13 non-synonymous SNPs in the RAD tags that were under strong selective pressure (S12 Table). Among them, the SNP locus in Tea_308203 was located in the unigene ‘Singletons23344’, which is homologous to *Arabidopsis* At5g66180, encoding an S-adenosyl-L-methionine (SAM)-dependent methyltransferase that catalyzes universal methylation. The SAM-dependent methyltransferase superfamily plays important roles in plant development [79], biosynthesis and modifying the structure of plant secondary metabolites [80], for example, the subfamliy of SAM-dependent N-methyltransferases has attracted the attention of tea researchers because it participates in the N-methylation steps in the biosynthesis of caffeine, a characteristic secondary metabolite in tea [73]. Moreover, the SNP locus in Tea_308825 is located in the unigene ‘Singletons120230’, which encodes a protein that is homolgous to the LRR receptor-like kinase 2 gene, which in turn shares a conserved structure and function with the known plant resistance genes that are involved in the innate immune system [81]. In rice, the rice blast resistance gene *Pik* (NBS-LRR gene), one of the five classical alleles located at the *Pik* locus on chromosome 11, has been characterized to be a younger allele emerging noly after rice domestication rather than evolving as a result of a duplication event [82]. These findings revealed the putative footprints of artificial selection on functional evolution during tea domestication.

The high heterozygosity of the tea genome was a barrier to the acquisition of detailed genomic information. In contrast to whole-genome sequencing approaches, the RAD-Seq approach focuses on single allelic differences or variations in smaller, more manageable portions of the genome that contain restriction sites and flanking sequences. Our results demonstrated the efficiency and cost-effectiveness of RAD-Seq technology in the generation of high-throughput genomic SNPs in *C*. *sinensis* and its wild relatives. This approach could easily be extended to include other restriction enzymes and identify additional SNPs to further enrich tea plant molecular genetic resources and improve our understanding of the effects of single nucleotide mutations on phenotypic traits.

The identified genomic SNPs first provided genome-wide information for the investigation of the genetic relationship and comparisons of the heterozygosities of the test cultivated and wild tea accessions in comparison with previous studies [15–16, 19–20, 22–26]. The SNPs evidently demonstrated the genetic divergence and variant heterozygosities between tea cultivars and wild relatives. The SNPs also provided the opportunity to glimpse the putative selective footprints on tea plants. Furthermore, we obtained usable information about the genic SNPs associated with gene functions for future research on the molecular mechanism of the distinct phenotypic traits of cultivated and wild tea plants and the improvement of tea breeding. Sampling is an important factor for genetic research. Considering the ambiguous genetic backgrouds of many wild species that are conserved from seed propagation in the National Tea Plant Germplasm Collection of China, all wild tea accessions used in the study were collected via natural field sampling. However, the sampling of wild accessions was limited because some wild resources have been partially destroyed by natural disasters and damage due to humans. The tea accession *C*. *taliensis* var. *bangwei* is the only semi-wild tea plant that has been reported [7] until now. Despite the relatively small population used in this study, the number of samples was comparable with those used in several molecular phylogenetic research papers focusing on *Pedicularis* [46], temperate bamboos [47] and Chinese bayberry [48] that used RAD-Seq technology. The methods for the identificaton of SNPs and genotyping were also similar to those used in these papers. Notably, expansion of the population size can increase the accuracy of SNP calling for inferring the genetic relationships at higher resolutions and provide a deeper comprehension of tea domestication. Therefore, there is an urgent need to increase field surveys of wild tea resources and increase the survival rate of cloned wild tea plants, which would benefit the enlargement of populations of wild tea resources. In future work, if we broaden the collection of *Camellia spp*. to more fully understand the phylogenetic relationships of the genus *Camellia* with SNPs at the genome-wide level, we will address the controversial taxonomy of the genus *Camellia*, decipher the origin and evolution of tea and benefit genetic breeding and improvements in tea.

In addition, the completement and high-quality of the reference database is another key factor for the bioinformatic analysis of SNPs. Although we used our previous tea transcriptome dataset from all tissues of *C*. *sinensis* cv. *Longjing43* [73] as the reference database, the tea plant genome should be the best reference database which can be used to identified more comprehensive SNP loci related to improtant traits such as plant defense and characteristic secondary metabolism. However, the genome complexity of the crop has encumbered us to obtain genomic information up to now. In the future, if the tea plant genome project are completed, we believe the tea plant genome data will prompt the biologic and genetic research in *Camellia* plants.

This study confirms that cultivated and wild tea plants are highly heterozygous presumably because of high self-incompatibility. Because the heterozygous rates of each accession were estimated based on shared SNP-associated genomic regions, the results can be used to compare of the relative heterozygosities of cultivated and wild tea genomes. It is important to note that RAD DNA fragments offer a reduced representation of the genome that contains only the restriction sites and their flanking sequences. The absolute nucleotide heterozygous rates across the entire genome cannot be extracted using this approach and can only be determined with whole genome sequencing. Accessions with lower heterozygosities are better suited to genome sequencing using NGS approaches.

## Materials and Methods

### Plant materials and DNA isolation

A total of 18 cultivated and wild tea accessions belonging to the section *Thea* of the genus *Camellia* were used in this study (Table 1). The nine cultivated tea accessions comprised three accessions of *C*. *sinensis* var. *assamica* (*Csa-1*, *Csa-2* and *Csa-3*) and six accessions of *C*. *sinensis* var. *sinensis* (*Css-1*, *Css-2*, *Css-3*, *Css-4*, *Css-5* and *Css-6*). *Csa-1* and *Csa-2* were sampled with the permission of the Menghai Agriculture Committee of the Yunnan province. *Csa-3* was developed from an ancient cultivated population in the Yunnan province using individual selective breeding methods and was sampled by the Tea Research Institute of the Yunnan Academy of Agricultural Science. Among the six *Css* accessions, *Css-1*, *Css-2*, *Css-3*, *Css-4* and *Css-5* are currently the main cultivars used in tea production, and these were sampled from three tea-producing regions in China; in contrast, *Css-6* is an F1 individual that resulted from a cross between *Csa-3* and *Css-5*. Permission for the tissue sampling of *Css-1* and *Css*-2 from agricultural plantations was obtained from Anhui Agricultural University. Sampling permission for *Css-3*, *Css-4*, *Css-5 and Css-6* was obtained from the Tea Research Institutes of the Academies of Agricultural Science in Anhui, Fujian and Yunnan, respectively. The other nine tea accessions are closely related to cultivated tea varieties and were sampled from trees in Yunnan province that are hundreds of years old. Among them, three accessions (*Ctl-1*, *Ctl-2* and *Ctl-3*) belong to *C*. *taliensis*, four (*Ccc-1*, *Ccc-2*, *Ccc-3* and *Ccc-4*) belong to *C*. *crassicolumna*, the *Ctg* accession belongs to *C*. *tachangensis*, and the *Ctb* accession belongs to *C*. *taliensis* var. *bangwei*, that is the only known semi-wild tea plant in the world based on evidence from morphological trait and karyotype analyses [7]. Permissions for the tissue samplings of *Ctl-1* and *Ctl-2*, *Ctl-3* and *Ctb*, and *Ctg* were obtained from the Menghai, Shuangjiang and Fuyuan Agriculture Committees in the Yunnan province, respectively. *Ccc-1*, *Ccc-2*, *Ccc-3* and *Ccc-4* were sampled with permission from the Tai Wai Mountain National Nature Reserve in the Yunnan province. All tissue sampling was performed under the supervision of local foresters, and the samples were used only for scientific research. The non-invasive sampling performed in this work did not affect the natural growth of the *Camellia* plants.

Buds and young leaves were randomly sampled from healthy young shoots of each accession and immediately frozen in liquid nitrogen. All samples were stored at −80°C until needed for DNA isolation. DNA samples were extracted from the buds and young leaves using a plant genomic DNA kit (Tiangen Biotech Co., China) following the manufacturer’s protocol. Residual RNA was removed from the genomic DNA by the treatment with RNase.

### RAD sequencing

RAD sequencing was performed as reported by Chutimanitsakun *et al* [44] with the exception that the restriction enzyme *EcoR*I (New England Biolabs) was used. Specific 4–8-bp nucleotide barcodes contained in the modified Illumina P1 adapters were used for sample tracking. To distinguish accession-specific barcodes from random single nucleotide differences caused by sequencing errors, the barcodes differed by at least two nucleotides between the different accessions. Subsequently, adapter-ligated DNA fragments were pooled and sheared to a mean size of 500 bp and separated with 2% agarose gel electrophoresis. Fragments of 350–500 bp were isolated using a MinElute Gel Extraction kit (Qiagen), treated with end-blunting enzymes, 3'-adenine overhangs were added, and the fragments were ligated with modified Illumina P2 adapters. Finally, the RAD-Seq libraries were enriched by PCR amplification and sequenced on an Illumina Hiseq 2000 (BGI, Shenzhen, China) using single-ended reads (50 bp) for each accession.

### RAD data analysis and SNP identification

The Illumina sequence reads were quality-filtered by removing the adapter sequences and reads containing greater than 50% low-quality bases (quality value ≤5). All reads were assigned to the tested accessions with unambiguous barcodes and the *EcoR*I recognition site AATTC (reads lacking unique barcodes and the specific sequence were discarded). The final clean reads were further trimmed to a uniform length of 41 nucleotides that included 5 nt of the *EcoR*I recognition site and 36 nt of potentially variable sequence.

Because a reference tea genome sequence is not currently available, the identification of SNPs was implemented *de novo* using Stacks software [58]. Briefly, the trimmed clean reads from each accession were aligned against each other, identical reads were clustered into one stack, and stacks with depths of coverage below 10-fold were discarded. Additionally, according to Emerson *et al* [83], if the sequencing reads in a particular stack were generated from repetitive sequence in the genome, the depth of coverage of the stack was much higher than the mean stack depth. Therefore, we removed the stacks with depths greater than 300-fold, and the remaining stacks were merged into a RAD tag locus after pairwise sequence alignment of the stacks that allowed for a maximum of one nucleotide mismatch between any two stacks. Within each accession, the genotype for each RAD tag locus at each nucleotide position was inferred, and a minimum 10-fold cut-off was used to classify the sites as homozygous when all of the bases were identical at a given nucleotide site. Nucleotide sites containing two alternative alleles (A1 and A2, which represent the first and second most frequently observed alleles with the highest and second depths, respectively) were defined as homozygotes when the ratio of the depths of the A2 and A1 was <0.05 (Depth_A2_ / Depth_A1_ <0.05) or as heterozygotes when Depth_A2_ / Depth_A1_ >0.1. Nucleotide sites with Depth_A2_ / Depth_A1_ value between 0.05 and 0.1 were discarded to minimize genotyping inaccuracies. After genotyping, a consensus sequence was assigned to each RAD tag locus.

Consensus sequences from each accession were compared across all accessions with a maximum of one mismatch allowed to generate putative SNP loci. After filtering, the RAD tag loci were genotyped for at least 14 of the 18 accessions (i.e., allowing a maximum of four accessions with missing sequence data at any given locus), and those containing only one bi-allelic SNP within the 36 nt of potentially variable sequence in each locus were retained to generate high-confidence SNPs.

### Phylogenetic analysis

To construct the phylogenetic tree, the genetic distances between the different accessions were calculated based on the high-confidence SNPs extracted from the RAD data. The *p*-distance, defined as D_ij_ between two accessions (*i* and *j*), was calculated using the following equation:

$${\text{D}}_{ij}=\frac{1}{L}\sum_{l=1}^{L}d_{ij}^{\left(l\right)}$$ (1)

where *L* is the length of the regions from which high-quality SNPs could be identified, and given that the allele at certain position was C/T, $d_{ij}^{\left(l\right)}$ was set to 0 if the genotypes of *i* and *j* were CC and CC, to 0.5 if the genotypes of *i* and *j* were CC and CT, and to 1 if the genotypes of *i* and *j* were CC and TT. The $d_{ij}^{\left(l\right)}$ value was set in the same manner used for the other five alleles. The phylogenetic tree was constructed using a neighbor-joining method based on a distance matrix calculated with MEGA5 [67], with bootstrap values at the default setting of 1000 trials.

### Principle component analysis

Principal component analysis was performed as previously reported [68]. The decomposition of the eigenvectors from the covariance matrix was performed with the R function Eigen, and the significances of the eigenvectors were further investigated with Tracey-Widom tests using the twstats program in the Eigensoft package [68].

### Genetic structure analysis

The analyses of the genetic structures of the tea accessions were performed using the program FRAPPE [69]. The individual ancestry proportion was calculated 10,000 times from a given number of inferred populations (*K*) based on a maximum likelihood algorithm [69]. The *K* values were set from two to six.

### Heterozygosity

The heterozygosity rates of the 18 tested tea accessions were evaluated by calculating the ratios of the numbers of heterozygous SNPs to the lengths of the shared SNP-associated genome fragments obtained from RAD sequencing in each accession using the following equation:

$$H=N_{hSNP}/L_{RAD-genome}$$ (2)

where *H* is the heterozygosity of a given tea accession, *N*_***hSNP***_ is the number of heterozygous SNPs identified in the 15,444 SNPs shared by 18 tea accessions, and *L*_***RAD-genome***_ is the total length of the RAD tags containing the 15,444 SNPs (41 nt of each RAD tag).

### Identification and functional analysis of genic SNP-associated genes

Among the 15,444 bi-allelic SNPs, the genic SNPs were identified based on the sequence alignments of the 15,444 SNP-associated RAD tag sequences against the tea transcriptome dataset (127,094 unigenes) from *C*. *sinensis* cv. *Longjing43* (sample ID: *Css-2*) [73] using the BLASTN algorithm of the NCBI-blast+—2.2.29 procedure (ftp://ftp.ncbi.nlm.nih.gov/blast/executables/blast+/2.2.29/). Strict thresholds were set with an E-value cut-off of 1e-5. A maximum of one mismatch was allowed, and alignment lengths above 80% and identities greater than 90% were required. For the gene annotations of the identified genic SNP-associated unigenes, the SNPs were compared with the *Arabidopsis* protein dataset using BLASTX with a strict E-value threshold of 1e-5. Functional classification according to GO terms [74] was performed by searching the top BLASTX hits against the NCBI *Arabidopsis* protein datasets using Blast2GO software (version 2.3.5) [75] with an E-value threshold of 1e-5. Among the genic SNPs based on the *C*. *sinensis* cv. *Longjing43* unigenes, we also identified the non-synonymous and synonymous substitutions from the coding sequences of the tea unigenes [73].

### Validation of SNP identification and genotyping

To experimentally validate the reliability of the SNP loci and genotyping of all of the 18 tested tea accessions, we randomly chose 50 identified genic SNP loci to perform 900 PCR amplifications and Sanger sequencing with SNP loci-specific primers. According to the best BLAST hits for the SNP loci-associated RAD tags with unigenes from the *C*. *sinensis* cv. *Longjing43* (sample ID: *Css-2*) transcriptome, we designed the SNP loci-specific primers according to the flanking sequences from the unigenes adjacent to the aligned regions using Primer Premier software (version 6.0; S9 Table). The primers that resulted in single bands of the expected sizes in *C*. *sinensis* cv. *Longjing43* were considered suitable for validating the genotyping of the 18 accessions. Genomic DNA was extracted and purified from young shoots using a DNeasy Plant Mini Kit (Aidlab, China). The PCR amplifications were performed in 25 μL of reaction volumes, containing 0.5 U Taq polymerase (TaKaRa), 5 nmol of each primer, and 10–30 ng DNA templates. The reactions were performed in a Bio-Rad Sequence Detection System with the following cycling parameters: 94°C for 3 min; 35 cycles of 94°C for 30 s; annealing at an optimum temperature for 30 s; 72°C for 30 s; and a final extension at 72°C for 10 min. The PCR products were sparated using agarose gel electrophoresis, purified and recovered using PCR purification kits and subjected to bi-directional sequencing on an ABI3730xl sequencer (Sangon Biotech Co. Ltd, China). At each SNP locus, sequences of all 18 accessions obtained by Sanger sequencing were aligned with SNP loci-associated RAD tag sequences using DNAman software.

### Diversity analysis and identification of putative domestication-related SNP loci

The average pairwise divergences between the cultivated (π _cultivated_) and wild groups (π _wild_) were calculated for each SNP locus with an in-house PERL script. According to the results from genetic relationship analysis, 6 *Css* accessions and 3 *Csa* accessions were included in the cultivated group, and the wild group was composed of all of the other 8 wild accessions except *Ctb*. We estimated the value of the loss of diversity (LOD) to detect the regions that were putatively under selection pressure [78] using the following equation:

$$\text{LOD}=1-\pi_{cultivated}/\pi_{wild}$$ (3)

The RAD tags comprising the SNP loci with significantly high LOD values that equaled 1 were identified as candidate regions that may have been affected by domestication, and tea unigenes related to fixed SNP loci were treated as putative domestication-related genes.

### Extraction and HPLC analysis of catechins

Catechins (flavan-3-ols), one kind of important secondary metabolites in tea, include non-galloylated catechins (epicatechin (EC), catechin (C), epigallocatechin (EGC), gallocatechin (GC)) and their gallate esters (mainly epicatechin gallate (ECG) and epigallocatechin gallate (EGCG)) [70]. Catechins were extracted from the samples according to the method described by Tai *et al* [84]. Briefly, 0.1 gram of freeze-dried sample was grounded into powder in liquid nitrogen, and then subjected to extraction with 3 mL 80% methanol using sonication for 10min at room temperature. The extractive was centrifuged at 6,000 rpm for 10 min for the supernatant. After the residues were re-extracted twice as described above, the supernatants were combined. The obtained supernatants were diluted with 80% methanol to a volume of 10 mL and filtered through a 0.22 μm organic membrane before HPLC analysis.

The filtered sample (10 μL) was injected into a Waters 2695 HPLC system equipped with a 2489 ultraviolet (UV)-visible detector for detection of the catechins contents in the extracts. The detection wavelength was set to 278 nm. A reverse-phase C18 column (Phenomenex 250 mm×4.6 mm, 5 micron) was used at 25°C. The samples were eluted at a flow-rate of 1 mL min^−1^ with the mobile phase containing 0.17% (v/v) acetic acid (A) in water, 100% acetonitrile (B), and the gradient elution was as follows: B 6% from 0 to 4 min, to 14% at 16 min, to 15% at 22 min, to 18% at 32 min, to 29% at 37 min, to 45% at 45 min, to 45% at 50 min, to 6% at 51 min and to 6% at 60 min. Samples from all tested accessions as mentioned above in RAD-Seq were analyzed in triplicate. The standards (purities > 98%) of gallic acid (GA), (+)-C, (−)-EC, (+)-GC, (−)-EGC, (+)-GCG, (−)-EGCG, and (−)-ECG were purchased from Shanghai Winherb Medical Science Co.,Ltd.,Shanghai, P.R. China.

## Conclusions

In this study, we applied RAD-Seq technology for the rapid and cost-effective discovery of 15,444 genomic SNPs from 18 tea accessions of *Camellia sinensis* and its wild relatives from the genus *Camellia* in the absence of prior genome sequences. The identified genomic SNPs have not only considerably increased the available molecular markers of *Camellia* but also provided comprehensive information about the genetic divergence and variant heterozygosities between cultivated and wild teas at the genome-wide level. These SNPs also provide the oppprtunity to glimpse putative selective footprints in tea plants. Genic SNPs related to functional genes, especially those involved in secondary metabolic processes, were identified and experimentally validated, which will aid future research on the molecular mechanism of distinct phenotypic traits of cultivated and wild teas. The genomic SNP data extend our knowledge of *Camellia* genomes, and the methods developed here can be applied to future genomics and phylogenomic studies and breeding programs for *Camellia* and other plants.

## Supporting Information

S1 Fig Transitions and transversions in the identified SNPs from the 18 tea accessions. (TIF)

S2 Fig Heterozygosity levels of six species/varieties in the section *Thea*. (TIF)

S3 Fig Gene Ontology classification of the non-synonymous SNP-associated tea unigenes. (TIF)

S1 Table RAD sequencing, quality filtering and *de novo* assembly of the 18 tested tea accessions. (DOC)

S2 Table SNPs identified in 15,444 RAD loci genotyped within at least 14 of 18 tea accessions. (XLSX)

S3 Table *De novo* SNP discoveries in 18 tea accessions. (DOC)

S4 Table Significant BLASTN hits of the 15,444 SNP-associated consensus sequences against the tea transcriptome dataset [73]. (XLSX)

S5 Table Top BLASTX hits of the genic SNP-associated tea unigenes against the *Arabidopsis* protein dataset. (XLSX)

S6 Table Gene Ontology IDs of the annotated genic SNP-associated tea unigenes. (XLSX)

S7 Table List of the main Gene Ontology categories and sub-categories of the annotated genic SNP-associated tea unigenes. (XLSX)

S8 Table Non-synonymous and synonymous SNPs identified based on the coding sequences of tea unigenes. (XLSX)

S9 Table Primers designed for the validation of the genotyping of the candidate SNP loci. (DOC)

S10 Table Validation of the SNP identification and genotyping of 50 candidate SNP loci in the 18 tea accessions. (XLSX)

S11 Table SNPs with the LOD values of 1 that were predicted to be under strong artificial selection. (XLSX)

S12 Table Genes with non-synonymous SNPs exhibiting strong selective signals. (XLSX)
