# Supplementary figures and images for: Genetic Divergence between Camellia sinensis and Its Wild Relatives Revealed via Genome-Wide SNPs from RAD Sequencing
Source: PLoS One. 2016 Mar 10;11(3):e0151424. doi: 10.1371/journal.pone.0151424 (PMC4786323; doi:10.1371/journal.pone.0151424)

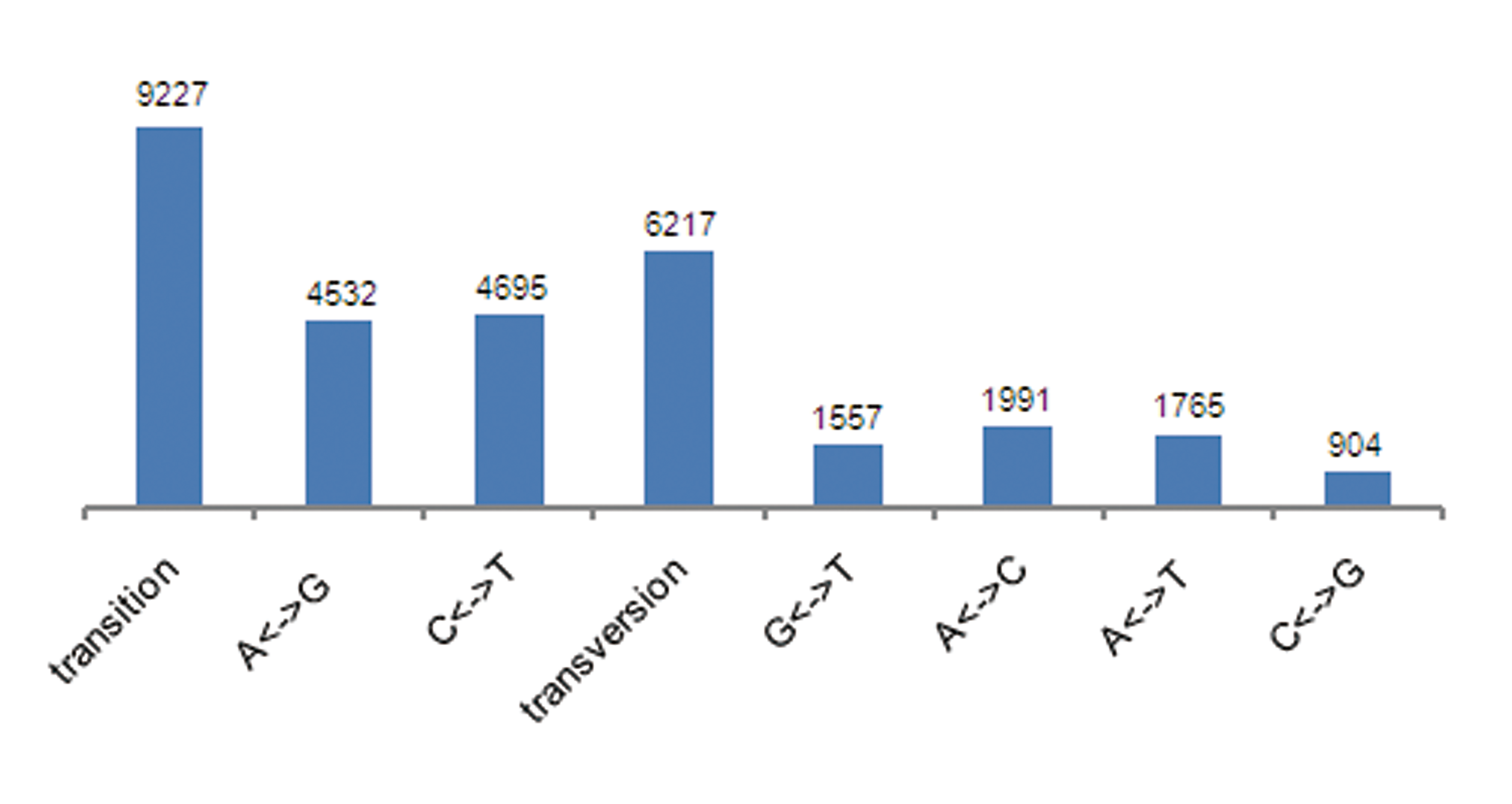

Supplement: S1 Fig — (TIF) [file pone.0151424.s001.TIF]

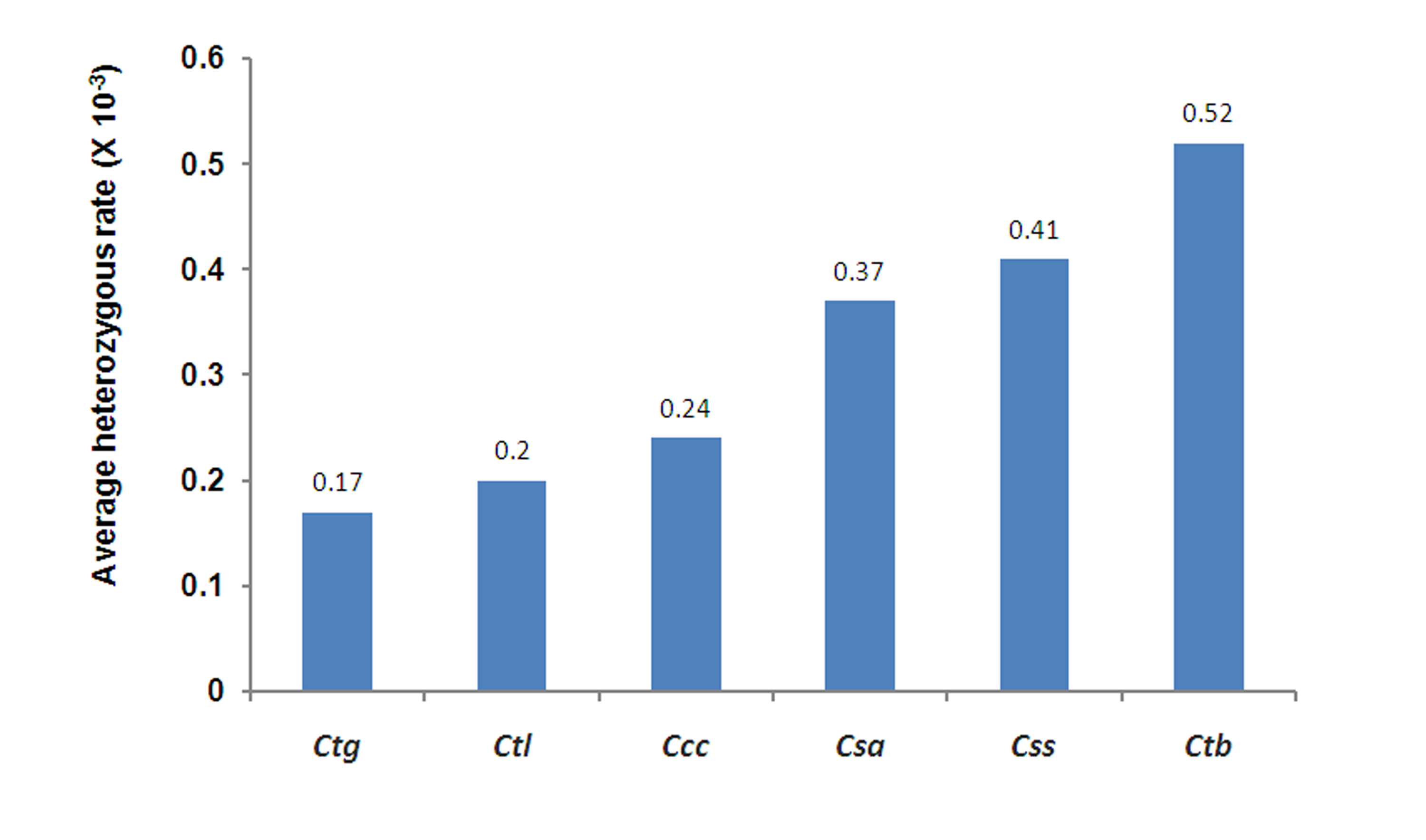

Supplement: S2 Fig — (TIF) [file pone.0151424.s002.TIF]

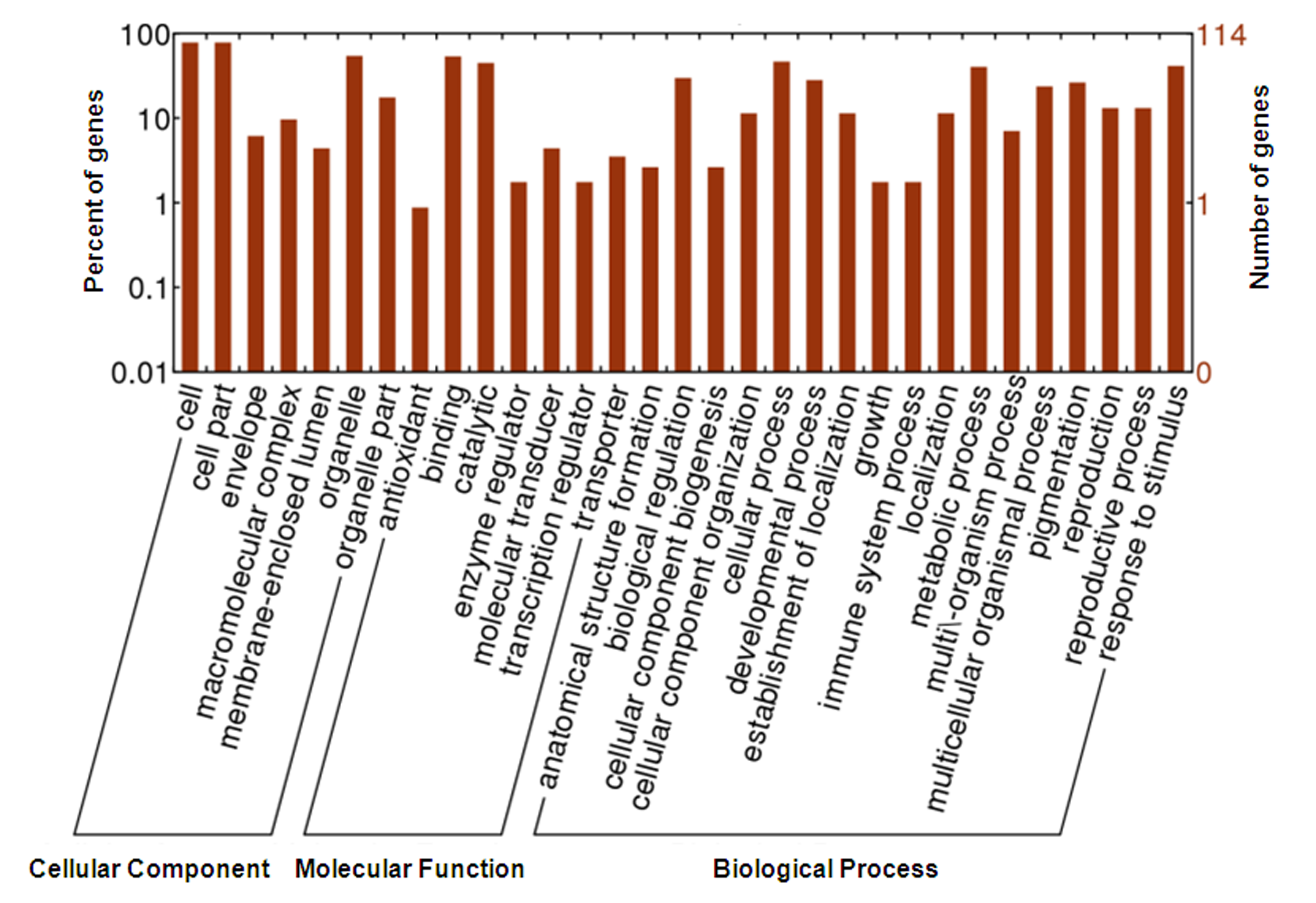

Supplement: S3 Fig — (TIF) [file pone.0151424.s003.TIF]
