# Supplementary material for: Genetic Divergence between Camellia sinensis and Its Wild Relatives Revealed via Genome-Wide SNPs from RAD Sequencing
Source: PLoS One. 2016 Mar 10;11(3):e0151424. doi: 10.1371/journal.pone.0151424 (PMC4786323; doi:10.1371/journal.pone.0151424)
Supplement: S1 Table — (DOC) [file pone.0151424.s004.doc]

**S1 Table RAD sequencing, quality filtering and data analysis of the 18 tested tea** accessions

| Sample ID | Raw read counts | Raw read length (bp) | Raw data (MB) | Clean read counts | Clean read length (bp) | Clean data (MB) | RAD tag loci counts | Average RAD tag loci depth (X) | RAD genome size (MB) | Heterozygous RAD tag loci counts | Heterzogous RAD genome size (MB) |
| --- | --- | --- | --- | --- | --- | --- | --- | --- | --- | --- | --- |
| *Ctl-1* | 67,006,106 | 44 | 2948.3 | 66,522,750 | 41 | 2727.4 | 955,115 | 69 | 39.2 | 285,519 | 11.7 |
| *Ctl-2* | 94,513,068 | 44 | 4158.6 | 93,121,036 | 41 | 3818.0 | 1,083,196 | 85 | 44.4 | 331,866 | 13.6 |
| *Ctl-3* | 42,434,338 | 45 | 1909.5 | 41,713,815 | 41 | 1710.3 | 666,577 | 62 | 27.3 | 194,406 | 8.0 |
| *Ccc-1* | 72,512,828 | 46 | 3335.6 | 71,768,605 | 41 | 2942.5 | 977,679 | 73 | 40.1 | 190,420 | 7.8 |
| *Ccc-2* | 103,915,572 | 42 | 4364.5 | 103,234,618 | 41 | 4232.6 | 1,015,113 | 101 | 41.6 | 290,947 | 11.9 |
| *Ccc-3* | 77,835,097 | 45 | 3502.6 | 77,316,481 | 41 | 3170.0 | 959,301 | 80 | 39.3 | 282,201 | 11.6 |
| *Ccc-4* | 68,516,886 | 42 | 2877.7 | 67,968,494 | 41 | 2786.7 | 880,433 | 77 | 36.1 | 263,601 | 10.8 |
| *Ctg* | 55,848,876 | 42 | 2345.7 | 55,433,017 | 41 | 2272.8 | 861,790 | 64 | 35.3 | 256,931 | 10.5 |
| *Ctb* | 85,806,140 | 43 | 3689.7 | 85,191,517 | 41 | 3492.9 | 1,115,146 | 76 | 45.7 | 342,117 | 14.0 |
| *Csa-1* | 98,062,202 | 42 | 4118.6 | 97,448,888 | 41 | 3995.4 | 1,055,366 | 92 | 43.3 | 287,560 | 11.8 |
| *Csa-2* | 61,523,860 | 44 | 2707.0 | 60,841,201 | 41 | 2494.5 | 1,033,238 | 58 | 42.4 | 319,709 | 13.1 |
| *Csa-3* | 67,750,677 | 43 | 2913.3 | 67,453,569 | 41 | 2765.6 | 1,151,695 | 58 | 47.2 | 354,488 | 14.5 |
| *Css-1* | 60,341,942 | 43 | 2594.7 | 59,950,933 | 41 | 2458.0 | 962,104 | 62 | 39.4 | 301,494 | 12.4 |
| *Css-2* | 78,222,073 | 46 | 3598.2 | 77,621,550 | 41 | 3182.5 | 979,045 | 79 | 40.1 | 285,251 | 11.7 |
| *Css-3* | 65,424,886 | 43 | 2813.3 | 61,469,083 | 41 | 2520.2 | 1,407,965 | 43 | 57.7 | 692,229 | 28.4 |
| *Css-4* | 73,606,937 | 45 | 3312.3 | 72,694,574 | 41 | 2980.5 | 1,034,911 | 70 | 42.4 | 311,771 | 12.8 |
| *Css-5* | 51,091,694 | 43 | 2196.9 | 50,196,872 | 41 | 2058.1 | 1,031,650 | 48 | 42.3 | 338,804 | 13.9 |
| *Css-6* | 80,694,966 | 42 | 3389.2 | 80,345,863 | 41 | 3294.2 | 1,119,819 | 71 | 45.9 | 345,435 | 14.2 |
| Total value | 1,305,108,148 | - | 56,775.6 | 1,290,292,866 | - | 52,902.0 | 18,290,143 | - | 749.9 | 5,674,749 | 232.7 |
| Mean value | 72,506,008 | - | 3,154.2 | 71,682,937 | - | 2,939.0 | 1,016,119 | 70 | 41.7 | 315,264 | 12.9 |
