# Supplementary material for: Genetic Divergence between Camellia sinensis and Its Wild Relatives Revealed via Genome-Wide SNPs from RAD Sequencing
Source: PLoS One. 2016 Mar 10;11(3):e0151424. doi: 10.1371/journal.pone.0151424 (PMC4786323; doi:10.1371/journal.pone.0151424)
Supplement: S3 Table — (DOC) [file pone.0151424.s006.doc]

**S3 Table *De novo* SNP discovery from the 18 tea accessions**

| Sample ID | Heterozygous SNP counts | Homozygous SNP counts | Missing SNP counts | Total used SNP counts | Total shared SNP counts | Missing rate (%) | Consensus sequence length (bp) | Average depth of SNPs (X) | Used RAD genome size (kb) | Heterozygous rate |
| --- | --- | --- | --- | --- | --- | --- | --- | --- | --- | --- |
| *Ctl-1* | 1,379 | 13,132 | 933 | 14,511 | 15,444 | 6.04 | 41 | 39.2 | 595.0 | 0.0023 |
| *Ctl-2* | 1,128 | 12,808 | 1,508 | 13,936 | 15,444 | 9.76 | 41 | 46.9 | 571.4 | 0.0020 |
| *Ctl-3* | 769 | 10,760 | 3,915 | 11,529 | 15,444 | 25.35 | 41 | 34.3 | 472.7 | 0.0016 |
| *Ccc-1* | 1,112 | 11,465 | 2,867 | 12,577 | 15,444 | 18.56 | 41 | 36.8 | 515.7 | 0.0022 |
| *Ccc-2* | 1,531 | 12,977 | 936 | 14,508 | 15,444 | 6.06 | 41 | 61.7 | 594.8 | 0.0026 |
| *Ccc-3* | 1,177 | 11,840 | 2,427 | 13,017 | 15,444 | 15.71 | 41 | 40.1 | 533.7 | 0.0022 |
| *Ccc-4* | 1,341 | 12,508 | 1,595 | 13,849 | 15,444 | 10.33 | 41 | 43.5 | 567.8 | 0.0024 |
| *Ctg* | 832 | 11,166 | 3,446 | 11,998 | 15,444 | 22.31 | 41 | 35.3 | 491.9 | 0.0017 |
| *Ctb* | 3,104 | 11,368 | 972 | 14,472 | 15,444 | 6.29 | 41 | 50.5 | 593.4 | 0.0052 |
| *Csa-1* | 1,336 | 12,124 | 1,984 | 13,460 | 15,444 | 12.85 | 41 | 51.6 | 551.9 | 0.0024 |
| *Csa-2* | 2,095 | 11,797 | 1,552 | 13,892 | 15,444 | 10.05 | 41 | 36.8 | 569.6 | 0.0037 |
| *Csa-3* | 2,927 | 11,721 | 796 | 14,648 | 15,444 | 5.15 | 41 | 39.1 | 600.6 | 0.0049 |
| *Css-1* | 1,348 | 10,651 | 3,445 | 11,999 | 15,444 | 22.31 | 41 | 32.8 | 492.0 | 0.0027 |
| *Css-2* | 1,541 | 12,161 | 1,742 | 13,702 | 15,444 | 11.28 | 41 | 47.1 | 561.8 | 0.0027 |
| *Css-3* | 4,927 | 9,952 | 565 | 14,879 | 15,444 | 3.66 | 41 | 34.6 | 610.0 | 0.0081 |
| *Css-4* | 1,864 | 12,603 | 977 | 14,467 | 15,444 | 6.33 | 41 | 42.0 | 593.1 | 0.0031 |
| *Css-5* | 1,746 | 11,791 | 1,907 | 13,537 | 15,444 | 12.35 | 41 | 27.9 | 555.0 | 0.0031 |
| *Css-6* | 2,895 | 12,173 | 376 | 15,068 | 15,444 | 2.43 | 41 | 46.3 | 617.8 | 0.0047 |
| Average value | 1,836 | 11,833 | 1,777 | 13,669 | 15,444 | 11.49 | 41 | 41.5 | 560.4 | 0.0032 |
