# Supplementary material for: Genetic Divergence between Camellia sinensis and Its Wild Relatives Revealed via Genome-Wide SNPs from RAD Sequencing
Source: PLoS One. 2016 Mar 10;11(3):e0151424. doi: 10.1371/journal.pone.0151424 (PMC4786323; doi:10.1371/journal.pone.0151424)
Supplement: S9 Table — (DOC) [file pone.0151424.s012.doc]

**S9 Table Primers designed for the validation of the genotyping of the candidate SNP loci**

| Tag_SNP ID | LJ43_Unigene ID | Primer ID | Primers (5'→3') | Tm (°C) | Product length (bp) |
| --- | --- | --- | --- | --- | --- |
| Tea_300658 | Singletons43716 | Tea_300658-F | GTGGAAGATAGCGGAGAA | 53.8 | 265 |
|  |  | Tea_300658-R | ATGAACAAGCAACTACCG | 51.4 |  |
| Tea_301446 | Singletons126133 | Tea_301446-F | GGACGAGCAACTAAACCAT | 55 | 292 |
|  |  | Tea_301446-R | AGTCACCTCAGGGACAAAT | 55 |  |
| Tea_301608 | Singletons39372 | Tea_301608-F | TGAGTCGGATTGATGGTTAT | 54.3 | 315 |
|  |  | Tea_301608-R | CAAAGGGTAGAAATGGCTGA | 56.4 |  |
| Tea_302550 | Singletons70758 | Tea_302550-F | GCATTGTAGCGACCAAAGAT | 56.4 | 274 |
|  |  | Tea_302550-R | AAGCACCAATCGTGTAGCA | 55 |  |
| Tea_302823 | Singletons114472 | Tea_302823-F | TGTATCAACCATCTCCCTC | 55 | 238 |
|  |  | Tea_302823-R | GGAAAGGCTCCAGAAACAA | 55 |  |
| Tea_303948 | Singletons44675 | Tea_303948-F | CAACACTCCCATTACCGA | 53.8 | 313 |
|  |  | Tea_303948-R | GCTTACTACCTCCTGCTA | 53.8 |  |
| Tea_303956 | Singletons8478 | Tea_303956-F | AGTAGCCGTTTGGATGGTA | 55 | 190 |
|  |  | Tea_303956-R | TTCGCAGATGATGTAGCAG | 55 |  |
| Tea_296903 | Singletons23067 | Tea_296903-F | AGTCTCTCTCCTGAACCA | 53.8 | 199 |
|  |  | Tea_296903-R | TCGCACCCTTGTTCTCTT | 53.8 |  |
| Tea_296913 | Singletons50402 | Tea_296913-F | TGGTCTCAGCCTACTACT | 53.8 | 290 |
|  |  | Tea_296913-R | CACACACGACAGGAAGCA | 56.3 |  |
| Tea_297534 | Singletons18304 | Tea_297534-F | AGAAAGTCGCTTCATCGC | 53.8 | 263 |
|  |  | Tea_297534-R | CTTCTCAGACCTCTCCAT | 53.8 |  |
| Tea_296219 | Singletons20034 | Tea_296219-F | ACCCATTGGTTTCTCTTCTC | 56.4 | 208 |
|  |  | Tea_296219-R | ATAGGGAAACGGAGAGTAAT | 54.3 |  |
| Tea_298380 | Singletons31795 | Tea_298380-F | TCACTCACAGCCCTAATCT | 55 | 246 |
|  |  | Tea_298380-R | TGTAATCGTCATAGTCGTC | 53 |  |
| Tea_298263 | Singletons19310 | Tea_298263-F | CAGTGATTTTCAACGATGG | 53 | 256 |
|  |  | Tea_298263-R | CTCATCACCAATCCCTCG | 56.3 |  |
| Tea_298271 | Singletons41697 | Tea_298271-F | CCTATTTGTTCAATGTCCC | 53 | 204 |
|  |  | Tea_298271-R | AGATGGACCAGTTGTTCG | 53.8 |  |
| Tea_298765 | Singletons122584 | Tea_298765-F | ACTCATTCCATCTGCGTT | 51.4 | 199 |
|  |  | Tea_298765-R | TTGCTGATGATTCCCTTC | 51.4 |  |
| Tea_299488 | Singletons14799 | Tea_299488-F | TAGATTACCGTTGGGGAC | 53.8 | 246 |
|  |  | Tea_299488-R | ATGTGATTCGGGGTTTGA | 51.4 |  |
| Tea_299891 | Singletons40470 | Tea_299891-F | TGCCAAAGGTCGGTTAGC | 56.3 | 206 |
|  |  | Tea_299891-R | GCAGAGTTATTCAGGGTCG | 57.5 |  |
| Tea_286507 | Singletons28730 | Tea_286507-F | CCCCTGATGGTTGCTCTT | 56.3 | 265 |
|  |  | Tea_286507-R | CACTTGGCTGCTGAGGAT | 56.3 |  |
| Tea_294945 | Singletons120065 | Tea_294945-F | TATGACCGTTGGCTATTTC | 53 | 292 |
|  |  | Tea_294945-R | GTTTTTGATGATTGAGCGTG | 54.3 |  |
| Tea_295982 | Cluster883_Consensus1 | Tea_295982-F | AGCACTTTCAACACAGGCA | 55 | 401 |
|  |  | Tea_295982-R | TCTCAAGATGGCTCAACC | 53.8 |  |
| Tag_SNP ID | LJ43_Unigene ID | Primer ID | Primers (5'→3') | Tm (°C) | Product length (bp) |
| Tea_294683 | Singletons46973 | Tea_294683-F | ACCAACGAGCACCGACTT | 56.3 | 398 |
|  |  | Tea_294683-R | GATTCAGAGCAACAAGCAA | 53 |  |
| Tea_294591 | Singletons50940 | Tea_294591-F | GAGCCTACGAATGGGAAGA | 57.5 | 218 |
|  |  | Tea_294591-R | AGATTGGTGATGGTGCCTGA | 58.4 |  |
| Tea_292013 | Singletons54798 | Tea_292013-F | GCACCTAAAGATTGGATTG | 53 | 308 |
|  |  | Tea_292013-R | GATGTGGGCAGATTACGC | 56.3 |  |
| Tea_280141 | Singletons27834 | Tea_280141-F | CGGAACTTGACTGTGCTAT | 55 | 258 |
|  |  | Tea_280141-R | CTCTGGCAGTTCACTATTTC | 56.4 |  |
| Tea_294868 | Singletons32892 | Tea_294868-F | GGGTCACTACCAATGATAAA | 54.3 | 166 |
|  |  | Tea_294868-R | TTTCCTAATGTCCCAGTC | 51.4 |  |
| Tea_304699 | Singletons10902 | Tea_304699-F | TTGAGGAAATGGGTAAGAC | 53 | 194 |
|  |  | Tea_304699-R | TGTTCTTGTCATCAGCCT | 51.4 |  |
| Tea_305283 | Singletons15331 | Tea_305283-F | GTTATTGAAGACCACGACAC | 56.4 | 210 |
|  |  | Tea_305283-R | CACAATGATGAGGAGGATGA | 56.4 |  |
| Tea_305298 | Singletons115421 | Tea_305298-F | AAGGGTCCTGTTCGTGATG | 57.5 | 332 |
|  |  | Tea_305298-R | AATGCCCGCTCCATACTG | 56.3 |  |
| Tea_305506 | Singletons53614 | Tea_305506-F | CTGTAATGTCTACGCTTGT | 53 | 246 |
|  |  | Tea_305506-R | ACCCTGACCAGCCAATAAA | 55 |  |
| Tea_306191 | Singletons7564 | Tea_306191-F | ATGTCAGTCCCGTCTCAG | 56.3 | 295 |
|  |  | Tea_306191-R | GTTGTTGCGACGACGATA | 53.8 |  |
| Tea_300576 | Singtons22067 | Tea_300576-F | TGGGAACCTTTCTGAACT | 51.4 | 215 |
|  |  | Tea_300576-R | GTGAATGTGGTATCTCCC | 53.8 |  |
| Tea_303052 | Singtons22068 | Tea_303052-F | TCACCTCTTCTCCCTTTC | 53.8 | 396 |
|  |  | Tea_303052-R | AGATTCTTGTGGGTTGTG | 51.4 |  |
| Tea_303755 | Singtons2015 | Tea_303755-F | CCCAAATGAACCTCCAAAC | 55 | 281 |
|  |  | Tea_303755-R | CTGGAGCTGATGGTGAGT | 56.3 |  |
| Tea_296329 | Singtons47964 | Tea_296329-F | GCTCAAGGAAAGTGGATT | 51.4 | 293 |
|  |  | Tea_296329-R | TTCCAAGGCTGATACTCG | 53.8 |  |
| Tea_306731 | Singtons37370 | Tea_306731-F | CTCGCTTCATTGGTAGTGT | 55 | 161 |
|  |  | Tea_306731-R | CAGGAAGGTTATGGGAGA | 53.8 |  |
| Tea_121721 | Singtons6739 | Tea_121721-F | GACCACAATCAGACCCAC | 56.3 | 236 |
|  |  | Tea_121721-R | AGCATTGGCTTACTCACA | 51.4 |  |
| Tea_122723 | Cluster1130_Consensus1 | Tea_122723-F | CTTCCAGGCTGCTTGTTC | 56.3 | 323 |
|  |  | Tea_122723-R | ATCTTTATGCTTCCACCAC | 53 |  |
| Tea_286606 | Singtons48896 | Tea_286606-F | CAACTGCCACAAGAATGAC | 55 | 242 |
|  |  | Tea_286606-R | ATCTTAAACAAGCACCACC | 53 |  |
| Tea_287134 | Singtons40931 | Tea_287134-F | TCACCGATAATCACAACC | 51.4 | 196 |
|  |  | Tea_287134-R | TCCACAATGCCAAGAACC | 53.8 |  |
| Tea_304239 | Singletons41458 | Tea_304239-F | CCTGCGTAGGTTACATCAA | 55 | 297 |
|  |  | Tea_304239-R | TCGGACCCTCCTTCTTTT | 53.8 |  |
| Tea_304596 | Singletons35348 | Tea_304596-F | GTTCACCAACCTCCACAA | 53.8 | 220 |
|  |  | Tea_304596-R | TCATATCAGCCATCTTGC | 51.4 |  |
| Tag_SNP ID | LJ43_Unigene ID | Primer ID | Primers (5'→3') | Tm (°C) | Product length (bp) |
| Tea_307241 | Singletons951 | Tea_307241-F | GCGAACAACCAAGTCAGT | 53.8 | 287 |
|  |  | Tea_307241-R | GCAGATGACCTTCCTGTATT | 56.4 |  |
| Tea_307743 | Singletons31369 | Tea_307743-F | GCAACAGGATTTGTCTAAG | 53 | 284 |
|  |  | Tea_307743-R | CATCTCAAGGTTTGGAAG | 51.4 |  |
| Tea_307759 | Singletons65645 | Tea_307759-F | AGGTTGGCTCATTCAGTG | 53.8 | 256 |
|  |  | Tea_307759-R | CTACAGTCATCTCATCCG | 53.8 |  |
| Tea_307787 | Singletons43458 | Tea_307787-F | GATTCGTGCTTGGCTTTC | 53.8 | 194 |
|  |  | Tea_307787-R | ATCCATCATTGGTCCCTC | 53.8 |  |
| Tea_307831 | Singletons43658 | Tea_307831-F | CAACGAGCACTTTGAGAG | 53.8 | 138 |
|  |  | Tea_307831-R | TACCTAAGACCTTCCGAT | 51.4 |  |
| Tea_307999 | Singletons43336 | Tea_307999-F | TTCACCTTCCCGTAATGC | 53.8 | 329 |
|  |  | Tea_307999-R | TACACCTCAACAGAGATGG | 55 |  |
| Tea_308203 | Singletons23344 | Tea_308203-F | TTTACTGAGCCCAACCACT | 55 | 226 |
|  |  | Tea_308203-R | ATTCTCCCTGTTTCCCAT | 51.4 |  |
| Tea_308204 | Singletons3926 | Tea_308204-F | GATTGCTTGAGGGCTAAC | 53.8 | 236 |
|  |  | Tea_308204-R | ACACCATCACTGAACTGC | 53.8 |  |
| Tea_308223 | Singletons28129 | Tea_308223-F | CCACCCATTACAGCATAC | 53.8 | 295 |
|  |  | Tea_308223-R | TTGGCTTCCCCGTTTCTAC | 57.5 |  |
